# Longitudinal study of surrogate aging measures during human immunodeficiency virus seroconversion

**DOI:** 10.18632/aging.101184

**Published:** 2017-02-23

**Authors:** Janice M Leung, Nick Fishbane, Meaghan Jones, Alexander Morin, Stella Xu, Joseph CY Liu, Julie MacIsaac, MJ Milloy, Kanna Hayashi, Julio Montaner, Steve Horvath, Michael Kobor, Don D Sin, P Richard Harrigan, SF Paul Man

**Affiliations:** ^1^ Centre for Heart Lung Innovation, St. Paul's Hospital, University of British Columbia, Vancouver, V6Z 1Y6, Canada; ^2^ Division of Respiratory Medicine, Department of Medicine, St. Paul's Hospital, University of British Columbia, Vancouver, V6Z 1Y6, Canada; ^3^ BC Centre for Excellence in HIV/AIDS, St. Paul's Hospital, University of British Columbia, Vancouver, V6Z 1Y6, Canada; ^4^ Centre for Molecular Medicine and Therapeutics, University of British Columbia, Vancouver, V5Z 4H4, Canada; ^5^ Department of Medicine, University of British Columbia, Vancouver, V6Z 1Y6, Canada; ^6^ Departments of Human Genetics and Biostatistics, University of California Los Angeles, Los Angeles, CA 90095, USA

**Keywords:** HIV, aging, methylation, telomere, seroconversion

## Abstract

Persons living with human immunodeficiency virus (HIV) harbor an increased risk of age-related conditions. We measured changes in telomere length and DNA methylation in the peripheral blood of 31 intravenous drug users, who were followed longitudinally with blood samples pre-HIV (T1), immediately post-HIV (T2; 1.9±1 year from T1), and at a later follow-up time (T3; 2.2±1 year from T2). Absolute telomere length measurements were performed using polymerase chain reaction methods. Methylation profiles were obtained using the Illumina Human Methylation450 platform. Methylation aging was assessed using the Horvath method. Telomere length significantly decreased between T1 and T2 (227±46 at T1 vs. 201±48 kbp/genome at T2, p=0.045), while no differences were observed between T2 and T3 (201±48 at T2 vs. 186±27 kbp/genome at T3, p=0.244). Methylation aging as measured by the age acceleration residual increased over the time course of HIV infection (p=0.035). CpG sites corresponding to *PCBP2* and *CSRNP1* were differentially methylated between T1 and T2 at a q-value <0.05. Telomere shortening and methylation changes can therefore be observed in the short-term period immediately following HIV seroconversion. Further studies to confirm these results in larger sample sizes and to compare these results to non-HIV and non-injection drug users are warranted.

## INTRODUCTION

With the benefit of combination antiretroviral therapy (cART), persons living with human immunodeficiency virus (PLWH) have survived to older ages [1, 2] with fewer opportunistic infections and AIDS-defining cancers [3, 4]. Despite these gains, the rise in the number of age-related conditions such as coronary artery disease [5], chronic obstructive pulmonary disease [6, 7], and non-AIDS-defining cancers [8] has sparked interest in how PLWH age. The precise nature of this heightened aging process, whether in fact accelerated or merely accentuated [9], is still unknown. PLWH appear to have shorter peripheral blood telomere lengths compared with uninfected individuals [10-12], yet whether this represents a gradual attrition over the course of HIV infection or an abrupt shortening during periods of acute illness and profound immuno-suppression has not been established. Recently, it has been shown in a cohort of cART-treated, virally suppressed PLWH that while telomere length, a surrogate marker of cellular aging, is shorter in PLWH compared with HIV-uninfected individuals, the slope of telomere length vs. age is no different between the two groups [11]. This might suggest that abrupt shortening does indeed occur early on in the course of disease, possibly at the period of intense immunosuppression related to acute HIV infection and prior to the institution of cART.

Identifying the timing of an aging trigger along the course of HIV infection has important scientific and clinical ramifications. At the very least, this allows further investigation into the biology of HIV aging to be situated in the appropriate time frame. Cellular changes observed within this time period provide important clues into the susceptibility of PLWH to age-related conditions. Shortening or preventing the onset of this time period may be one strategy that can improve outcomes in an aging HIV-infected population. Such investigations, however, require longitudinal sampling from subjects before and after HIV seroconversion. In this study, we examined surrogate peripheral blood aging markers in a cohort of injection drug users (IDU) followed longitudinally before and after acquiring HIV, aiming to 1) identify when age acceleration might occur in HIV and 2) describe potential key biologic pathways perturbed during he HIV seroconversion period. Useful biomarkers of aging, according to criteria adopted by the American Federation of Aging Research, are required to fulfill the following objectives: 1) predict the rate of aging; 2) reflect a biologic process associated with aging; 3) be able to be tested repeatedly in an individual without harm; and 4) translate from animals to humans [13]. Our choices of surrogate aging measures, both of which meet these criteria, focus on two distinct mechanisms, the first relating to replicative senescence by measuring peripheral blood telomere length and the second relating to age-associated methylation changes via a DNA methylation clock [14, 15]. Shortened telomere length has been shown in some studies to predict mortality [16], age-related diseases [17], and has been widely used as a biomarker of aging. A recent study also demonstrated that the DNA methylation clock is a useful biomarker for detecting accelerated aging effects due to HIV infection [18], but the behavior over time of this biomarker is not yet known. Here we demonstrate for the first time that changes in surrogate aging biomarkers may be observed shortly after HIV infection.

## RESULTS

### Study cohort

Demographics for the 31 patients enrolled in the study are provided in Table 1. The cohort had a mean age of 35.8 years and 48% were male. Nearly all patients (90%) had concurrent hepatitis C infection. The samples were collected between 1999 and 2004; by T3, only seven (22%) were on cART. The mean time intervals (± standard deviation [SD]) were 1.9 ± 1 years between T1 and T2 and 2.2 ± 1 years between T2 and T3.

**Table 1:** Demographics of the study cohort

Characteristic	Result (n=31)
Age (years ± SD)	35.8 ±10.1
Male Sex (%)	15 (48.4%)
Ever Smoker (%)	28 (90.3%)
CD4 Count at T2 (cells/mm^3^ ± SD)	386 ± 213
CD4 Count at T3 (cells/mm^3^ ± SD)	273 ± 150
Viral Load at T2 (copies/mL ± SD)	96,432 ± 164,340
Viral Load at T3 (copies/mL ± SD)	58,461 ± 55,575
On cART at T3 (%)	7 (22.6%)
Hepatitis C	28 (90%)
Current Homelessness	15 (48.4%)
Injection Drug Use Within Last 6 Months	25 (81%)
Previous Incarceration	25 (88.2%)
History of Physical Abuse	27 (87.0%)
History of Sexual Abuse	15 (48.4%)
Heavy Alcohol Use Within Last 6 Months (>4 drinks/day)	5 (16.1%)

Abbreviations: SD – standard deviation; cART – combination antiretroviral therapy

### Absolute telomere length measurements

**Figure 1:**
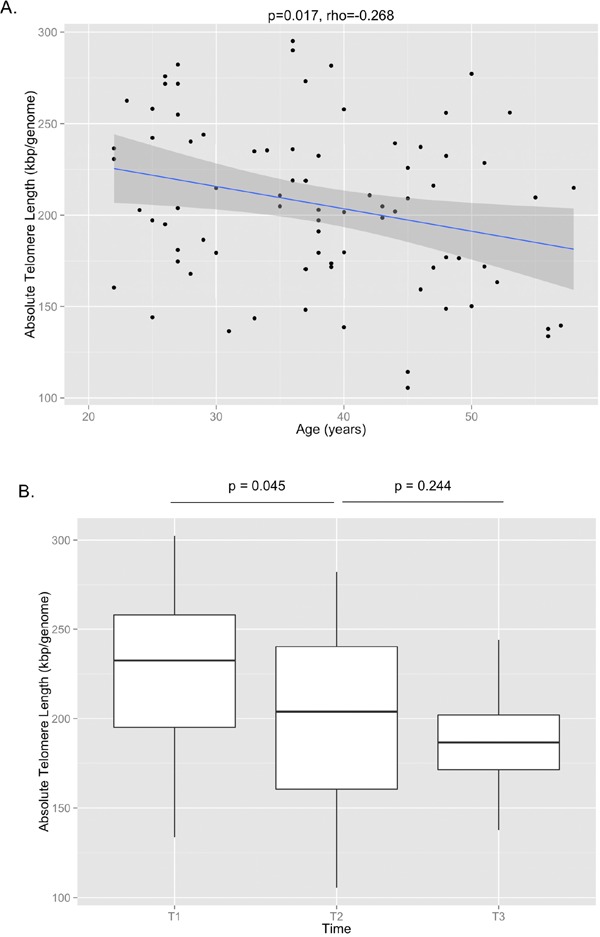
(**A**)The correlation between absolute telomere length and age is shown, demonstrating that shorter telomere lengths are observed with older age (p=0.017, Pearson's rho=-0.268). (**B**) Absolute telomere length measurements are shown for T1, T2, and T3. By paired t-test, there was a significant decrease in telomere length between T1 and T2 (p=0.045), but no significant change between T2 and T3 (p=0.244).

29 paired samples from T1 and T2 and 17 paired samples from T2 and T3 were included in the final analysis after samples from two individuals between T1 and T2 and two individuals between T2 and T3 failed qPCR runs. The correlation between telomere length and age is shown in Figure 1A (p=0.017, Pearson's rho=−0.268). The mean telomere length (± SD) was 227 ± 46 kbp/genome at T1, 201 ± 48 kbp/genome at T2, and 186 ± 27 kbp/genome at T3 (Figure 1B). Paired t-test analysis showed that the telomere length at T2 was significantly shorter than T1 (p=0.045), but that the telomere length at T3 was not significantly different from T2 (p=0.244). There were no significant differences at the T3 time point between the telomere lengths of subjects on cART and those not being treated with cART, nor were there any differences between those with detectable or undetectable viral loads or between those with CD4 counts <200 cells/mm^3^ or ≥200 cells/mm^3^.

### DNA methylation age

**Figure 2:**
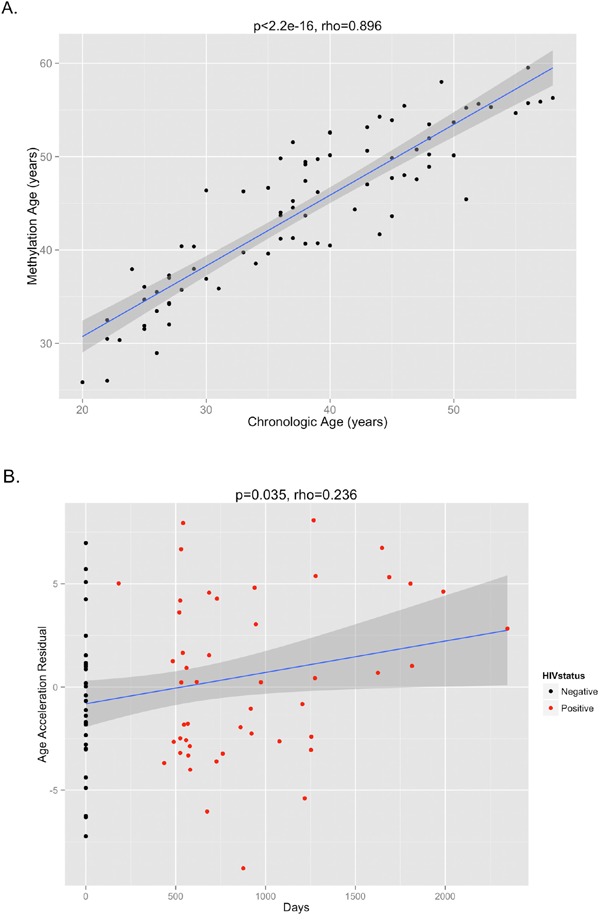
(**A**) The correlation between DNA methylation age and chronologic age from all subjects inclusive of all time points is shown, demonstrating a high correlation between the two measures (p<2.2e-16, Pearson's rho=0.896). (**B**) The age acceleration residual (greater positivity on this scale represents more advanced methylation age in relation to chronologic age) increases over the time course of HIV infection (p=0.035, Pearson's rho=0.236). Black dots represent HIV-negative time points (T1) while red dots represent HIV-positive time points (T2 and T3).

The DNA methylation age was calculated for each subject at the various time points. The correlation between DNA methylation age and chronologic age is shown in Figure 2A. To determine the magnitude of age acceleration over the duration of HIV infection, the correlation between the age acceleration residual and time point in days along the course of the study is shown in Figure 2B. Epigenetic age acceleration was positive correlated with days since HIV infection (p=0.035, Pearson's r=0.236) which shows that HIV infection accelerates the biological aging rate of blood.

### DNA methylation profiling

**Figure 3:**
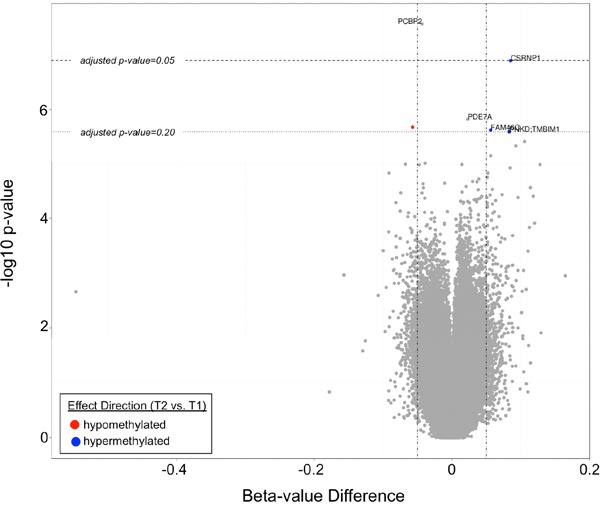
A volcano plot demonstrating differentially methylated CpG sites in T2 vs. T1. Two CpG sites (corresponding to *PCBP2* and *CSRNP1*) had q-values <0.05, while four additional CpG sites (corresponding to *PDE7a*, *FAM46C*, *PNKD:TMBIM1*, and an unknown gene) had q-values <0.20.

CD4 and CD8 T cell type proportion percentages at T1, T2, and T3 are provided in Supplementary Figure 1A and 1B. CD4 cell percentages decreased significantly between T1 and T2 (p<0.001), whereas CD8 cell percentages increased significantly between T1 and T2 (p<0.001). There were no significant changes in either CD4 or CD8 cell percentages between T2 and T3. Cell percentages of monocytes, NK cells, granulocytes, and B cells were not significantly different between the three time points (Supplementary Figure 1C, 1D, 1E,

The top differentially methylated CpG sites between T1 and T2 are listed in Table 2. After adjustment for cellular composition of blood, there were two CpG sites differentially methylated between T1 and T2 with a q-value <0.05. These were cg07151565, corresponding to *PCBP2* (hypomethylated in T2 vs. T1, q-value=0.012), and cg23654821, corresponding to *CSRNP1* (hyper-methylated in T2 vs. T1, q-value=0.028). There were four additional CpG sites that were differentially methylated with a q-value <0.20. These sites are shown in a volcano plot in Figure 3. One CpG site was found to be differentially methylated between T2 and T3, how-ever only at a q-value of 0.065. This was cg07926733 (corresponding gene currently unknown). The mean beta-value difference (T3 vs. T2) was 0.064.

**Table 2:** Differentially methylated CpG sites (T2 vs. T1)

CpG Site	Gene	Beta-Value Difference	q-value
cg07151565	*PCBP2*	−0.043	0.012
cg23654821	*CSRNP1*	0.085	0.028
cg10252135	*PDE7a*	0.023	0.180
cg21149466	Unknown	−0.057	0.180
cg02854554	*FAM46C*	0.056	0.180
cg25353281	*PNKD;TMBIM1*	0.083	0.180

### Pyrosequencing validation

Pyrosequencing validation was carried out for five of the six differentially methylated CpG sites between T1 and T2 (cg10252135 was not validated due to the small beta-value difference of 0.023 which was within the range of pyrosequencing error) and for the one differentially methylated CpG site between T2 and T3. Spearman correlation plots for the six CpG sites are shown in Supplementary Figure 2. Four CpG sites had Spearman correlation rho values near or above 0.8, indicating agreement between the two methods (cg02854554, cg23654821, cg25353281, and cg07926733).

## DISCUSSION

In this study investigating longitudinal blood samples of IDUs who subsequently contracted HIV, we found an acute telomere shortening signal in the immediate post-seroconversion period. Telomere length subsequently stabilized in the post-seroconversion period with no significant changes observed between the T2 and T3 time points. The magnitude of telomere length shortening between T1 and T2, almost 30 kbp/genome in an average time span of two years or less (equivalent to a loss of ~650 base pairs/year), was a striking finding reflecting the severity of replicative senescence in HIV. A previous study that used the same method of telomere length measurement across a large general population sample showed that an equivalent drop in telomere length would have taken roughly 40 years [11]. On average, leukocyte telomere length is expected to decline only by approximately 25 base pairs per year in the general population [19]. No doubt, the VIDUS population, all IDUs, represent a specific high-risk group of individuals, but the additional stress of acute HIV infection may account for the extra telomere length loss. Future prospective studies evaluating the equivalent drop in telomere length for IDUs who did not contract HIV would be key to determining whether these findings are HIV-specific.

Telomere length, however, reflects only one particular aspect of the complex process of aging. Moreover, it is an imperfect reflection, carrying only a correlation of 0.3 with chronologic age [19]. Studies that have evaluated the association between telomere length with mortality have also yielded conflicting results [20]. For these reasons, we chose to measure aging by an additional method. While telomere shortening is a surrogate measure of the limits of cellular replication and of oxidative stress, DNA methylation changes over time represent the loss of methylation fidelity through cell division, a process known as “epigenetic drift” [21]. Overall patterns of methylation changes suggest that methylation tends to increase in promoter regions and decrease in non-island regions with age [22]. Recently developed methylation clocks, constructed based on these principles, can thus serve as additional evidence of aging in HIV. We observed greater deviations of the DNA methylation age from expected trajectories the further subjects were from their HIV diagnosis date. It is important to note, though, that the DNA methylation aging clock reflects a separate process of senescence from telomere shortening, with at best only a weak correlation between the two different measures [23]. In our data, we do not observe a significant correlation between epigenetic age acceleration and telomere length (p=0.60, rho=-0.06, Supplementary Figure 3) which shows that the epigenetic clock relates to a biological process that is distinct from that of telomere attrition.

By using a genome-wide DNA methylation platform, we performed a preliminary investigation into the unique biology of the HIV peri-seroconversion period. Methylation changes between T1 and T2 were subtle, with an average beta-value difference in CpG sites between the two time points not exceeding 10%, yet still revealed interesting genes that might serve as the target for future mechanistic work. The most significant changes were observed at CpG sites associated with the *PCBP2* and *CSRNP1* genes, which had q-values <0.05. *PCBP2*, found to be hypermethylated following seroconversion, encodes for poly(RC) binding protein 2, which has been demonstrated to be a translational coactivator of a number of viruses including polio [24] [25], norovirus [26], and hepatitis C [27, 28]. Notably, it has also been reported to play a role in HIV-1 gene expression [29]. Depletion of PCBP2 through siRNA results in increased expression of HIV-1 Gag and Env protein, for instance. *CSRNP1*, hypomethylated following seroconversion, encodes for cysteine-serine-rich nuclear protein 1 which through the Wnt signaling pathway has been shown to have a tumor suppressive function [30]. While there are no reported associations with HIV infection to our knowledge in the literature, the increased risk borne by PLWH for both AIDS-related and non-AIDS-related malignancies suggests this may be a relevant gene for investigating oncologic risk in this population. Genes found to be differentially methylated between T1 and T2 at lesser statistical significance included *PDE7a* (involved in cAMP degradation) [31], *FAM46C* (involved in the enhancement of viral replication) [32], *PNKD* (involved in familial paroxysmal nonkinesigenic dyskinesia) [33], and *TMBIM1* (involved in vascular remodeling through matrix metalloproteinase 9) [34]. As far as we are aware, none of these have previously been reported to have associations with HIV infection. Further validation of these targets, particularly with additional HIV-negative samples, is necessary at this point.

This study has several limitations which should be noted. First, without concurrent RNA or protein samples, DNA methylation alone cannot tell us the direction of gene expression or protein transcription. While genes methylated at their promoter CpG islands have traditionally been thought to be transcriptionally silent, this is no longer considered an exact rule [35]. Therefore, despite significant associations between HIV seroconversion and *PCBP2* and *CSRNP1* we are unable to determine whether these genes are up- or down-regulated, only that it is possible their expression may be altered by their methylation status. Second, the changes we observe in telomere length and DNA methylation with HIV seroconversion are limited here to IDUs, a unique population at a high risk for additional infections and health problems such as bacterial endocarditis and hepatitis C. Whether similar changes occur in non-IDUs contracting HIV are observed is unknown. Such investigations are hampered by the fact that very few cohorts exist that have longitudinally tracked high-risk populations prior to and following HIV infection. It should be noted that in a previous analysis of HIV-infected patients, telomere length was not significantly affected by Hepatitis C status (data not shown). Third, DNA methylation is highly associated with cell type composition. We have used a statistical method to deconvolute the cell composition of our whole blood samples; however, these methods can only partially account for the many cell subtypes that exist in peripheral blood [36]. Currently, statistical deconvolution methods have not included subsets of NK cells or myeloid cells that may also contribute to the overall methylation signature. Finally, the small sample size of our study is a limitation and certainly further work in larger cohorts would be key to strengthening these findings. In particular, expansion of the cohort to include IDUs who remained HIV-negative throughout the study period would be important to determine whether the findings of this study are specific to the HIV or IDU populations. We were also underpowered in this study to determine whether aging is more pronounced in those with more severe HIV, as marked by higher viral loads and lower CD4 cell counts. Subgroup analyses in a larger cohort would be important to pursue to investigate this hypothesis.

The results of this study suggest that the time period shortly after HIV seroconversion may be critical in the aging process of PLWH. Given that this is a time where profound changes occur in the immune system, with rapid destruction of CD4 T cells and proliferation of CD8 T cells, targeting this interval with immediate cART initiation may be one therapeutic intervention that can mitigate downstream aging complications. Such a theory warrants further prospective study and may be imperative in a population whose demographics are quickly aging.

## MATERIALS AND METHODS

### Study cohort

Subjects were enrolled from the Vancouver Injection Drug Users Study (VIDUS), a prospective cohort study of >1,000 IDUs in the Vancouver area that began in 1996 [37] (University of British Columbia Research Ethics Board Approval Number H01-50086). Enrollment criteria into the study included: 1) injection of illicit drugs at least once during the previous month, 2) residence in the Greater Vancouver area, and 3) ability to provide written informed consent. VIDUS participants were tested every six months for HIV and hepatitis C. Subjects included in this analysis had to have tested positive for HIV during follow-up with available peripheral blood samples prior to and after HIV seroconversion. Peripheral blood cell pellets were frozen at −70 degrees Celsius prior to use. 31 subjects were enrolled with pre- (T1) and post-seroconversion (T2) samples, with 19 of these subjects further pro-viding an additional third blood sample (T3) approximately two years after the immediate post-seroconversion period.

### Absolute telomere length measurement

Genomic DNA from peripheral blood cell pellets was harvested using the Qiagen DNeasy Blood & Tissue Kit (Qiagen, Venlo, the Netherlands). Absolute telomere length was measured by quantitative PCR consistent with methods outlined by O'Callaghan and Fenech [38]. Briefly, standard curves were generated from known quantities of synthesized oligomers of telomere (*TEL*) DNA [(TTAGGG)14] and single copy reference gene (*36B4*) DNA [CAGCAAGTGGGAAGGTGTAATCCG TCTCCACAGACAAGGCCAGGACTCGTTTGTACCCGTTGATGATAGAATGGG] (Sigma-Aldrich, St. Louis, MO). Sample telomere DNA length was then assessed based on the ratio of telomere DNA length to *36B4* DNA length as obtained from their respective standard curves. DNA from a short telomere cell line (*HEK293*) and a long telomere cell line (*K562*) (ATCC, Manassas, VA) were used as inter-experimental plate controls [39]. The telomere lengths measured using this method reflect an average length across the population of cells included in the sample. Samples were run in triplicate using the ABI ViiA 7 Real Time PCR System (Applied Biosystems, Foster City, CA). All samples were concurrently run to avoid significant batch effects. Paired t-tests were performed to compare telomere lengths at T1, T2, and T3 with p-values <0.05 (two-tailed tests) considered significant.

### DNA methylation profiling

DNA extracted from the peripheral blood cell pellets was bisulfite-converted using the EZ DNA Methylation™ Kit (Zymo, Irvine, CA). This step converts unmethylated cytosine residues to uracil while leaving methylated cytosine residues intact. DNA methylation profiles were then obtained using the Illumina Infinium Methylation 450K assay, which interrogates 485,512 cytosine-guanine (CpG) sites spanning 99% of RefSeq genes, with an average of 17 CpG sites per gene [40]. In total, 96% of all CpG islands in the genome are assessed. Beta-values (the ratio of the methylated probe intensity to the overall intensity, ranging from 0 indicating all unmethylated to 1 indicating all methylated) are calculated for each CpG site and converted to M-values (the log2 ratio of the intensity of the methylated probe versus unmethylated probe) for statistical analyses [41]. These data were normalized using functional normalization which corrects for technical variation and is recommended for data sets where global methylation changes are expected [42]. CpG sites were filtered for detection quality and probe binding specificity [43].

### DNA methylation age

We used the DNA methylation age-based biomarker of aging from Horvath because a) its accurate measurement of age across tissues is unprecedented [14]; b) it has been found to be useful for studying aging effects in HIV infection [18]; c) it is prognostic for all-cause mortality [15, 44]; d) it correlates with measures of cognitive and physical fitness in the elderly [45]; e) it has been used in many applications including Down syndrome [46], obesity [23], lifetime stress [47], and Parkinson's disease [48]; and f) it is applicable to populations of all ages, not just the elderly [49, 50].

Here, we calculated the DNA methylation age for each sample according to the Horvath algorithm (http://labs.genetics.ucla.edu/Horvath/dnamage/) [14]. This method uses the weighted regression of 353 CpG sites on the Illumina 450K platform to calculate a methylation age. The age estimate (referred to as DNAm age or epigenetic age) is calibrated so that it is in units of years. The measure of epigenetic age acceleration was defined as the residual from regressing DNAm age on chronological age. By definition, this measure of age acceleration is not correlated with chronological age. Greater positivity in the age acceleration residual is indicative of faster age acceleration. The correlation between the age acceleration residual and duration of time along the study was calculated using a Pearson's correlation test. P-values <0.05 were considered significant.

### Differentially methylated CpG sites

Because methylation profiles can be affected by cell type proportions [51], we used a deconvolution method for calculating the percentage of CD4 T cells, CD8 T cells, natural killer cells, monocytes, B-cells, and granulocytes in whole blood samples based on the methylation signature [52]. Differences in normalized methylation M-values between T1 and T2, T2 and T3, and T1 and T3 were modeled using a linear regression framework, adjusting for the differences in the cellular composition of peripheral blood between time points:

Methylation difference ~ intercept + CD4 T cell-difference + CD8 T cell-difference + natural killer cell-difference + monocyte-difference + B-cell-difference + granulocyte-difference

The coefficient of interest for which a t-statistic p-value is calculated is the intercept, the null hypothesis being that the methylation difference (represented by the intercept) between time points is 0. This inference was implemented using the limma algorithm [53]. CpG sites with false discovery rate (FDR) q-values <0.05 were considered to be differentially methylated with a high degree of confidence; however, given the small sample size, CpG sites with q-values <0.20 (considered differentially methylated with a medium degree of confidence) were also identified and retained for pyrosequencing validation.

### Pyrosequencing validation

CpG sites considered to be differentially methylated between time points at a q-value <0.20 were validated using bisulfite pyrosequencing methods. Further details on pyrosequencing methods and primer design are provided in the Supplementary Materials. Reproducibility was assessed between pyrosequencing and the Illumina 450K normalized beta-values using Spearman's rank correlation.

### Statistical program

All statistical analyses were performed using R version 3.2.1.

## SUPPLEMENTAL MATERIALS AND METHODS AND FIGURES


