# Supplementary material for: Longitudinal study of surrogate aging measures during human immunodeficiency virus seroconversion
Source: Aging (Albany NY). 2017 Feb 23;9(3):687–97. doi: 10.18632/aging.101184 (PMC5391226; doi:10.18632/aging.101184)
Supplement: Supplementary file 1 [file aging-09-687-s001.pdf]

SUPPLEMENTARY MATERIAL

Pyrosequencing Methods

PyroMark Assay Design 2.0 (Qiagen, Inc.) software was used to design the bisulfate pyrosequencing assay covering the targets regions. DNA was subjected to bisulfite conversion using the EZ DNA Methylation Kit (Zymo Research). HotstarTaq DNA polymerase kit (Qiagen, Inc.) was used to amplify the target regions using the biotinylated primer set with the following PCR conditions: 15 minutes at 95°C, 45 cycles of 95°C for 30s, 58°C for 30s, and 72°C for 30s, and a 5 minute 72°C extension step. Streptavidin-coated sepharose beads were bound to the biotinylated-strand of the PCR product and then washed and denatured to yield single-stranded DNA. Sequencing primers were introduced to allow for pyrosequencing (Pyromark™ Q96 MD pyrosequencer, Qiagen, Inc.).

Pyrosequencing Primers

The following primers were used for pyrosequencing validation:
ID: 1 (cg07926733)
PCR Forward: F1 AGTTAAGTTAGAGTAGTATTGG ATTATAGT
PCR Reverse: R1 Biot CCTATCTCCCTTAAATTCTT AAAACT
Seq Primer: S1 Fwd TAGAGGGAGAGAGGT

ID: 2 (cg07151565)
PCR Forward: F1 AGTTGTTAGTTTTGGTTAGTTAT TTATAAT
PCR Reverse: R2 Biot AACCAAATTTCTTTACCCTT TTTTC
Seq Primer: S2 Fwd ATAGTGTTGGTGGGG

ID: 3 (cg23654821)
PCR Forward: F4 GGAGGAGGAAGTAGAGTTATT ATAT
PCR Reverse: R3 Biot ATAAACTAACAAACCTCA ACCTAATCTC
Seq Primer: S7 Fwd ATGTAAGTTGTGTGAATTAT TT

ID: 4 (cg21149466)
PCR Forward: F1 GTTATTAAAGGTGGATGTGTA TAGAAAA
PCR Reverse: R1 Biot AAAAACACATTCAAATCC CTAAATCT
Seq Primer: S1 Fwd TGTAAGTTATAGTATTAGAG AAGT

ID: 5 (cg02854554)
PCR Forward: F1 GTGGGTTAGGAGATTGAATTA GTTT
PCR Reverse: R1 Biot AAACTCATTTCCTTACCAA TTTACTCA
Seq Primer: S17 Fwd GGTTTAGTTGTTTTTTG

ID: 6 (cg25353281)
PCR Forward: F1 GGTGGGAAGGGAGATATTAATG
PCR Reverse: R1 Biot CCCATTCCACACAACTACTAT
Seq Primer: S1 Fwd AGTTTTTATTTTTGTTTGTAAT GAT

A.

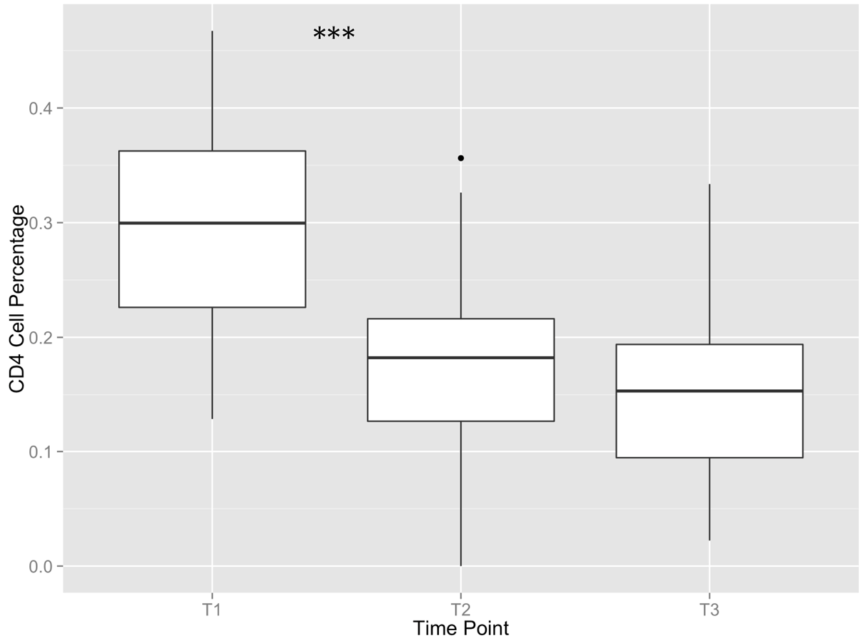

B.

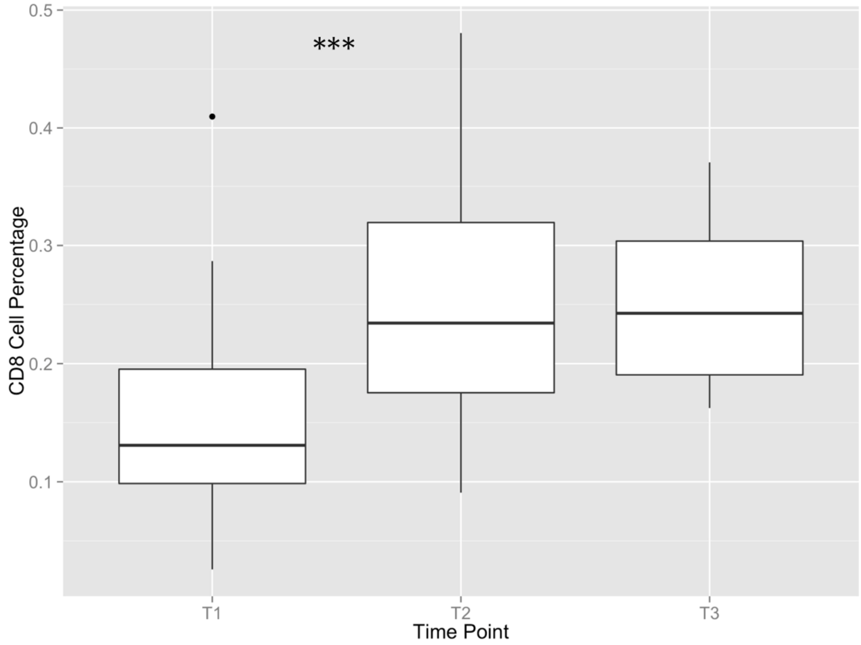

Supplementary Figure 1AB. Deconvolution methods by the Houseman-Jaffe algorithm yielded the cell proportions of (**A**) CD4 cells, (**B**) CD8 cells. Paired t-tests were performed to compare T1 vs. T2 and T2 vs. T3. There were statistically significant differences in the percentage of CD4 and CD8 cells between T1 and T2 (*** signifiying p<0.001). There were no statistically significant differences in the percentage of CD4 and CD8 cells between T2 and T3, nor were there statistically significant differences between any of the time points for monocytes, natural killer cells, granulocytes, and B cells.

C.

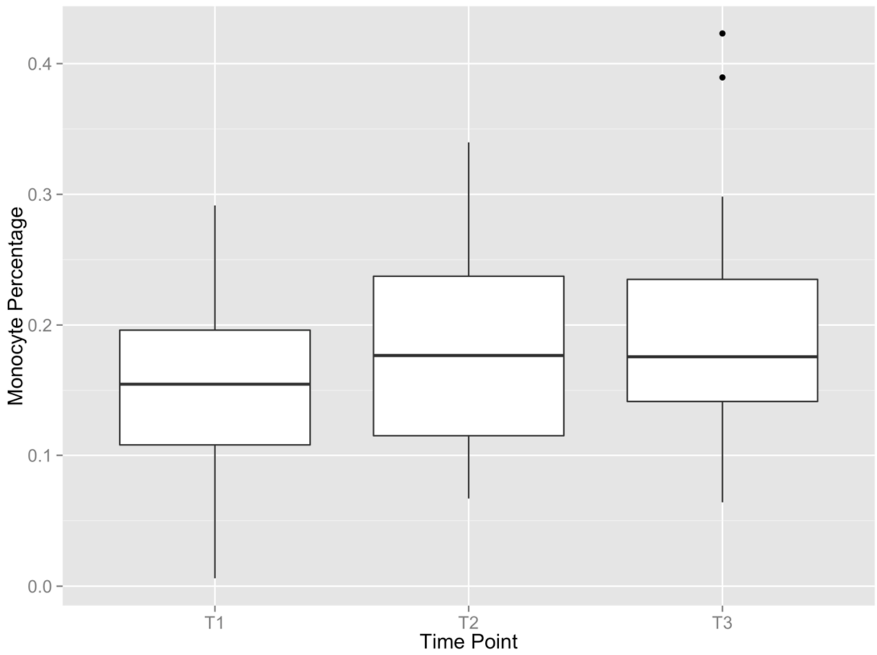

D.

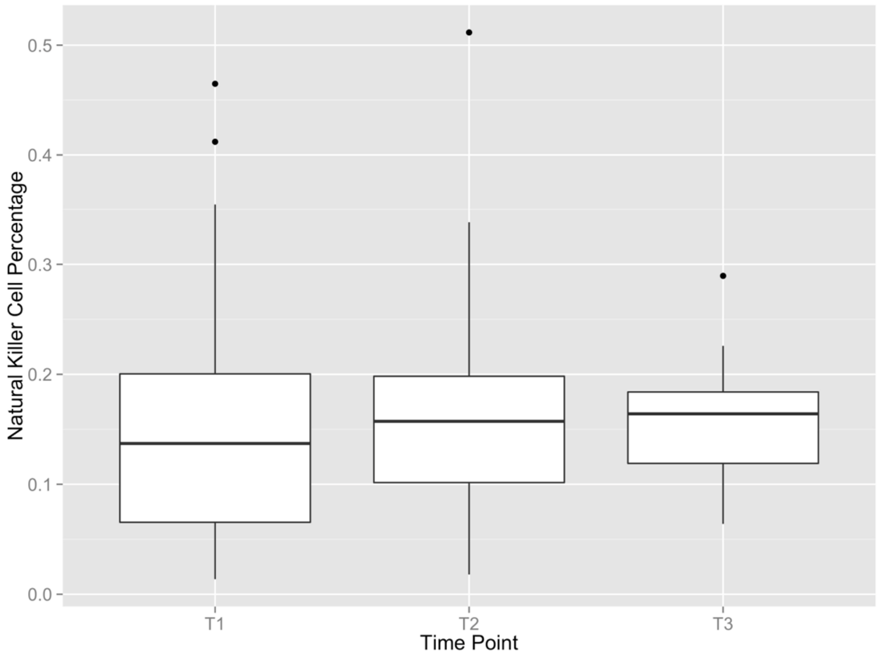

Supplementary Figure 1CD. Deconvolution methods by the Houseman-Jaffe algorithm yielded the cell proportions of (**C**) monocytes, (**D**) natural killer cells. Paired t-tests were performed to compare T1 vs. T2 and T2 vs. T3. There were statistically significant differences in the percentage of CD4 and CD8 cells between T1 and T2 (*** signifiying $p<0.001$). There were no statistically significant differences in the percentage of CD4 and CD8 cells between T2 and T3, nor were there statistically significant differences between any of the time points for monocytes, natural killer cells, granulocytes, and B cells.

E.

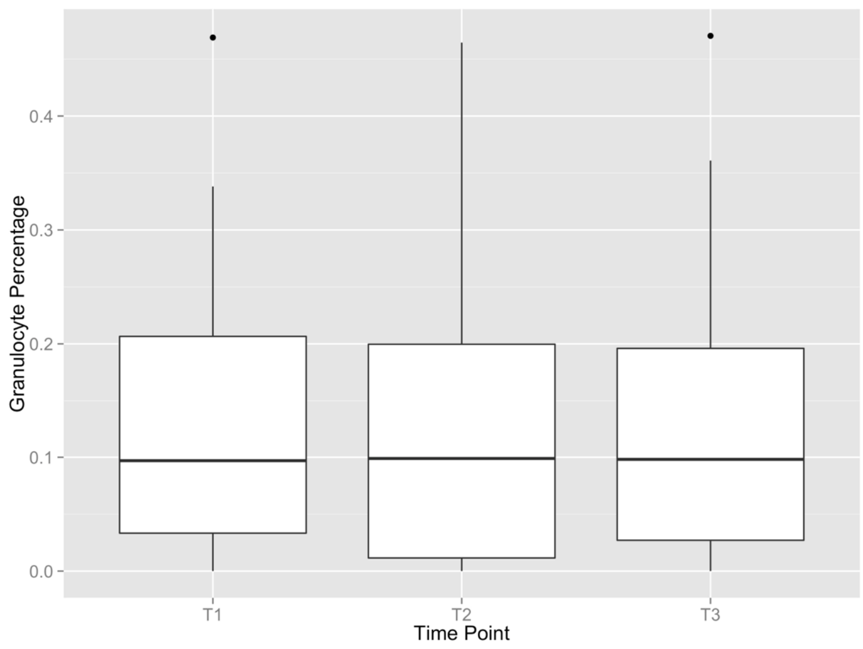

F.

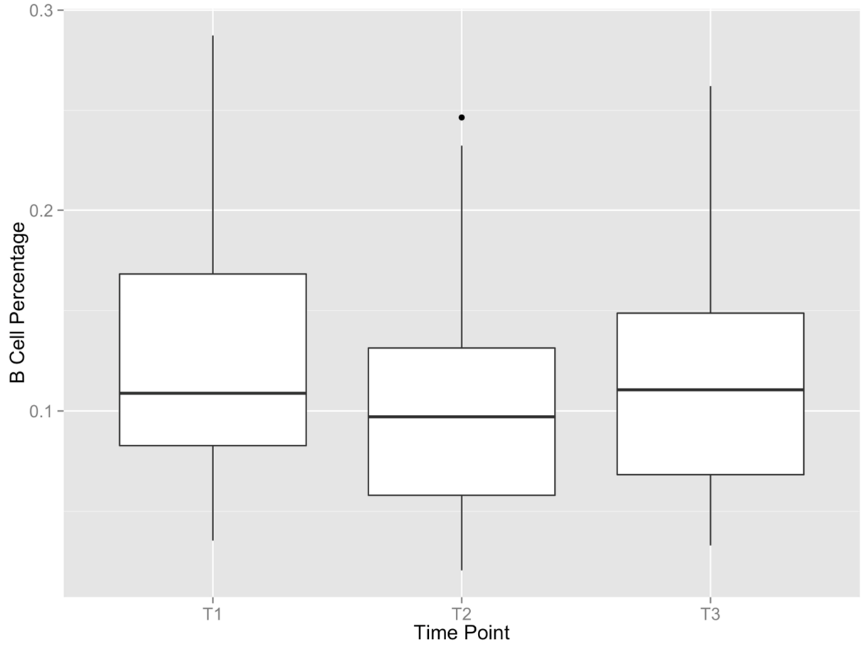

Supplementary Figure 1EF. Deconvolution methods by the Houseman-Jaffe algorithm yielded the cell proportions of (**E**) granulocytes, and **(F**) B-cells. Paired t-tests were performed to compare T1 vs. T2 and T2 vs. T3. There were statistically significant differences in the percentage of CD4 and CD8 cells between T1 and T2 (*** signifiying p<0.001). There were no statistically significant differences in the percentage of CD4 and CD8 cells between T2 and T3, nor were there statistically significant differences between any of the time points for monocytes, natural killer cells, granulocytes, and B cells.

A.

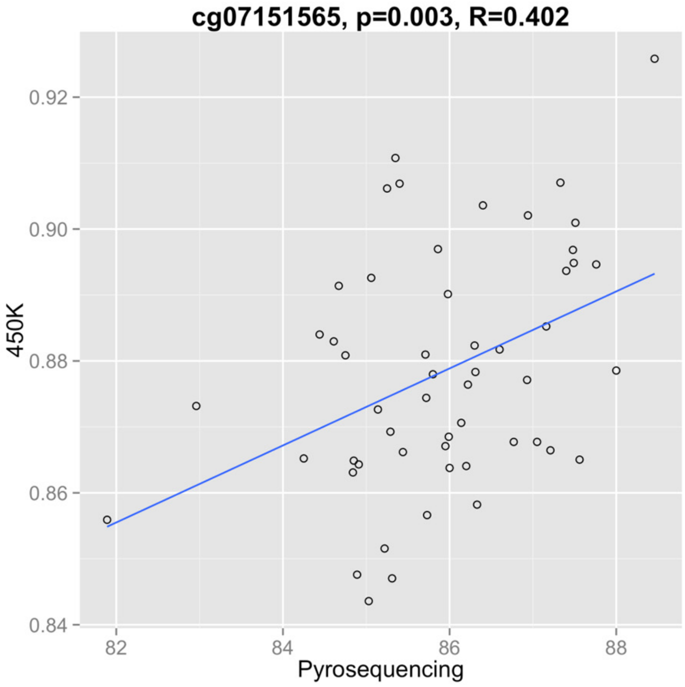

B.

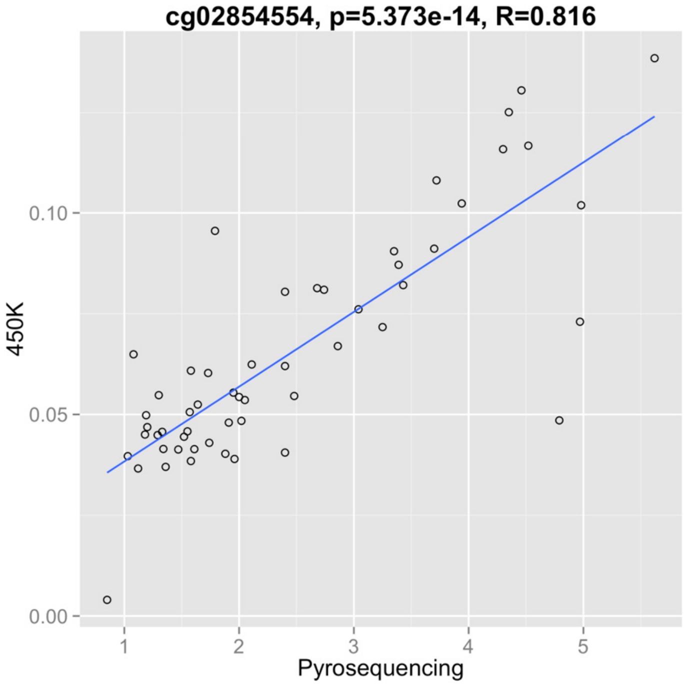

Supplementary Figure 2AB. Spearman correlation plots for 450K methylation beta-values vs.pyrosequencing methylation beta-values. (**A**) cg07151565, (**B**) cg02854554.

C.

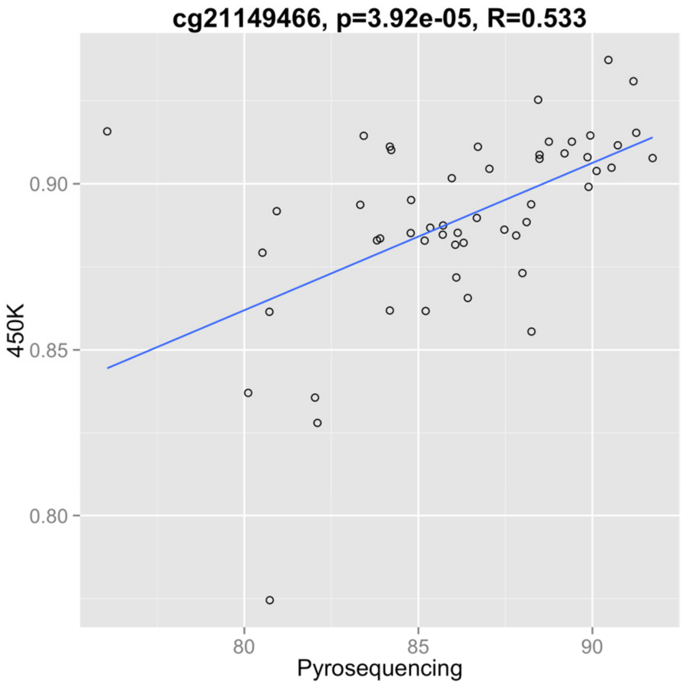

D.

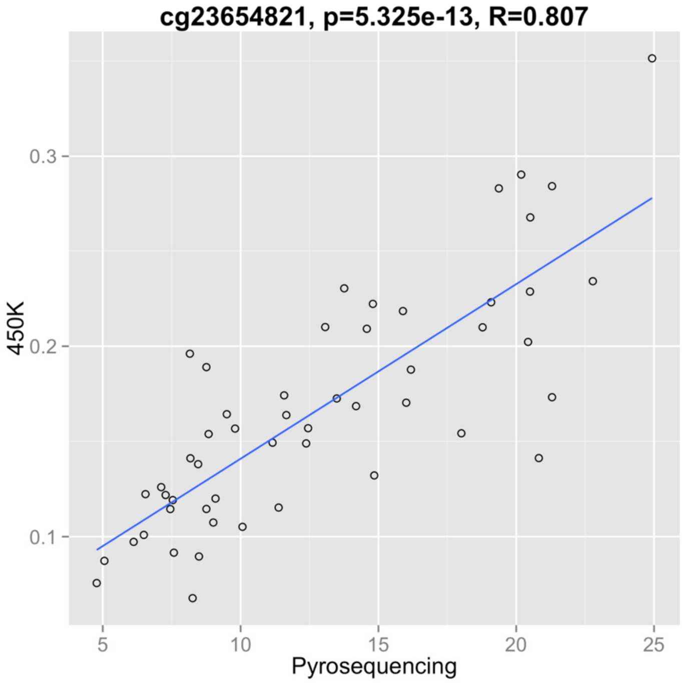

Supplementary Figure 2CD. Spearman correlation plots for 450K methylation beta-values vs.pyrosequencing methylation beta-values. (**C**) cg21149466, (**D**) cg23654821.

E.

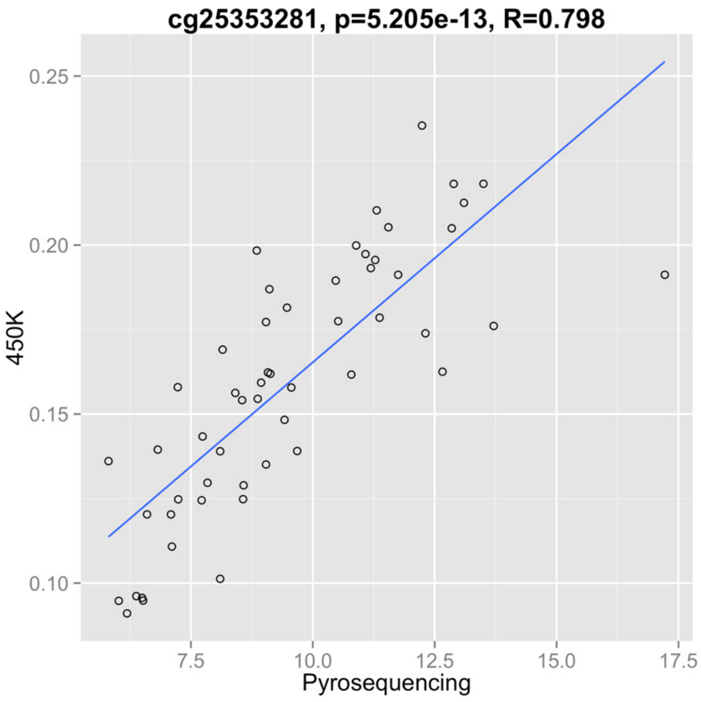

F.

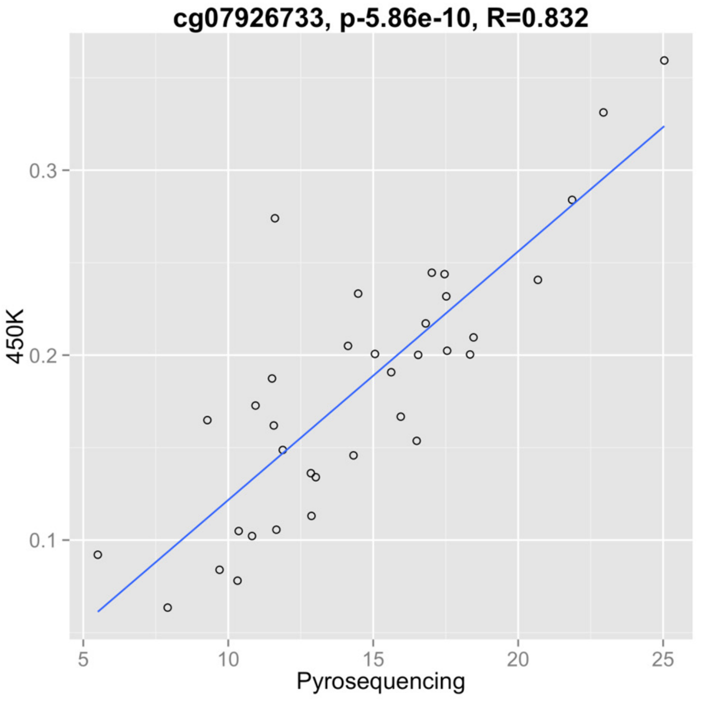

Supplementary Figure 2EF. Spearman correlation plots for 450K methylation beta-values vs.pyrosequencing methylation beta-values. (**E**) cg25353281, (**F**) cg07926733.

A.

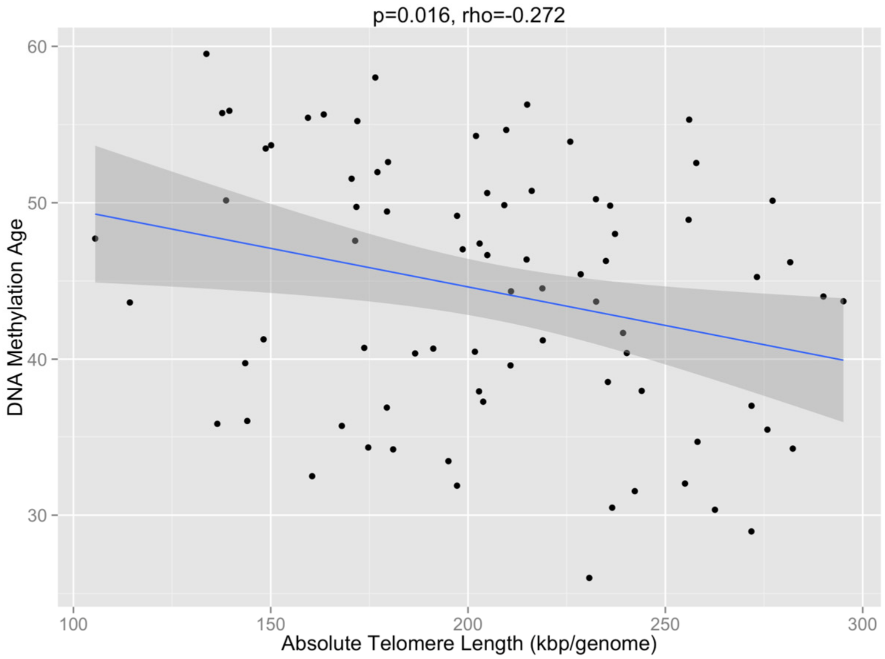

B.

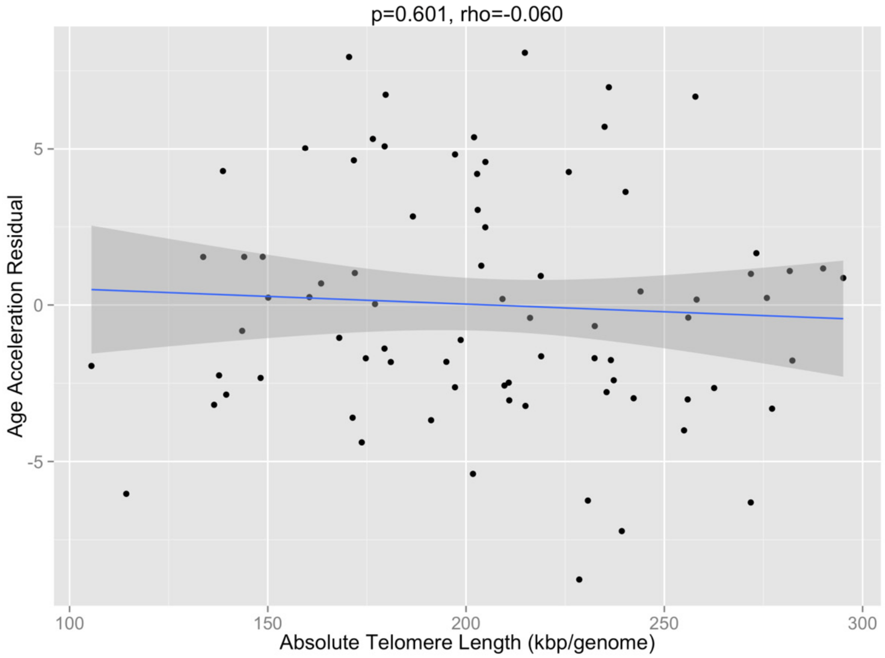

Supplementary Figure 3. Correlation plots between DNA methylation age and telomere length (Figure 3A) and between the age acceleration residual and telomere length (Figure 3B). These demonstrate very weak correlations between methylomic aging changes and telomere length.
